# The Spawns of Creative Behavior in Team Sports: A Creativity Developmental Framework

**DOI:** 10.3389/fpsyg.2016.01282

**Published:** 2016-08-26

**Authors:** Sara D. L. Santos, Daniel Memmert, Jaime Sampaio, Nuno Leite

**Affiliations:** ^1^Research Center in Sports Sciences, Health Sciences and Human Development, CreativeLab Research Community, University of Trás-os-Montes and Alto DouroVila Real, Portugal; ^2^Institute of Cognitive and Team/Racket Sport Research, German Sport University CologneCologne, Germany

**Keywords:** exploratory behavior, deliberate play, physical literacy, nonlinear pedagogy, expertise

## Abstract

Developing creativity in team sports players is becoming an increasing focus in sports sciences. The Creativity Developmental Framework is presented to provide an updated science based background. This Framework describes five incremental creative stages (*beginner, explorer, illuminati, creator*, and *rise*) and combines them into multidisciplinary approaches embodied in creative assumptions. In the first training stages, the emphasis is placed on the enrollment in diversification, deliberate play and physical literacy approaches grounded in nonlinear pedagogies. These approaches allow more freedom to discover different movement patterns increasing the likelihood of emerging novel, adaptive and functional solutions. In the later stages, the progressive specialization in sports and the differential learning commitment are extremely important to push the limits of the creative progress at higher levels of performance by increasing the range of skills configurations. Notwithstanding, during all developmental stages the teaching games for understanding, a game-centered approach, linked with the constraints-led approach play an important role to boost the tactical creative behavior. Both perspectives might encourage players to explore all actions possibilities (improving divergent thinking) and prevents the standardization in their actions. Overall, considering the aforementioned practice conditions the Creativity Developmental Framework scrutinizes the main directions that lead to a long-term improvement of the creative behavior in team sports. Nevertheless, this framework should be seen as a work in progress to be later used as the paramount reference in creativity training.

## Introduction

The relationship among creativity development and sport participation remains unarticulated. Creative behavior is an extremely valued disposition in everyday life (Runco, 2014), as well as in sport performance (Memmert, 2015b). Sports scientists are recognizing the importance of increasing research focused in the development of creativity in sport. Nowadays, there are many obstacles that restrain the creative potential, such as the lack of street sport, unadjusted training, mechanization of play, decrease of the game enjoyment and a narrow game knowledge. Moreover, the current sport systems are not aligned in order to supply these necessities. In fact, a rigid linear environment prevails and players are constantly overwhelmed by a lack of adaptability during the game. Although strategies can be planned and trained in advance, all dynamic interactions have a level of uniqueness to them and may even be unexpected. The resolution of competitive situations will always vary when taking into account the environmental influences. It is vital that the training environment reflects the real setting full of unpredictability, thus it is necessary to support the players' creativity. Creative solutions are of crucial importance to sport success, talent development and selection system and the key is let it blossom during the early years. Indeed, there is great potential in creative moments, which can lead the team toward outstanding performances. It appears that creative players make the difference, as they bring the unforeseeable into the game and disrupt the opponents (Memmert, 2013, 2015a).

Although there have been several empirically proven principles fostering tactical creativity driven by Memmert (2015a), little is known regarding the incorporation into age depending training programs specifically designed to develop and stimulate creative behavior in team sports within different kinds of expertise and/or levels. In this sense, the current article integrates the latest research in sport science and creativity into a developmental framework, in an attempt to provide a common ground for fostering tactical creativity across childhood and the junior age span. The Creativity Developmental Framework (CDF) highlights the idea that creativity depends on the mastery of thinking and sporting skills. For this reason, the creative behavior relies on distinct emerging patterns according to the players' progression in sport settings. Thus, it is mandatory to establish a supportive and rewarding environment for creative ideas to appear. To ensure an optimal development of creative behavior, the CDF mainly distinguishes five incremental training stages, that account for enhancing specific creative components: (a) *Beginner* stage (2–6 years); (b) *Explorer* stage (7–9 years);(c) *Illuminati* stage (10–12 years); (d) *Creator* stage (13–15 years); and (e) *Rise* stage (over 16 years). The presented stages are general guidelines created as a possible continuum of creativity boost during the early ages, however neither all players will follow this sequential path, adopting a more erratic, and distinct development.

**Figure 1:**
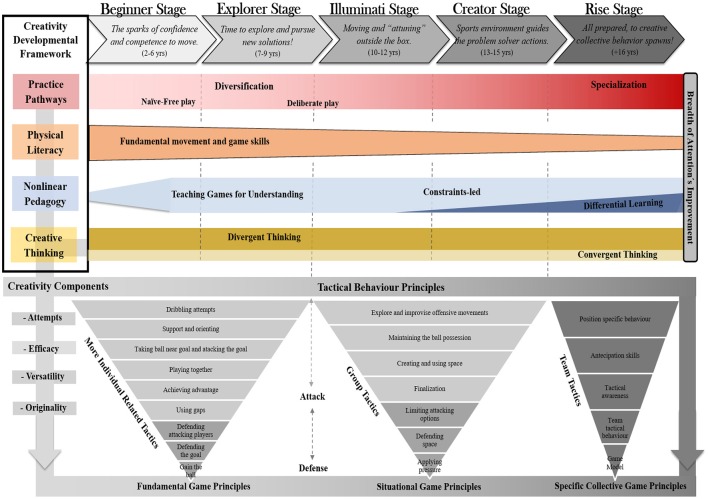
**The Creativity Developmental Framework structure supports the creative behavior in team sports**. Representation of the Creativity Developmental Framework structure: 1) Developmental creativity stages: (a) beginner; (b) explorer; (c) illuminati; (d) creator; (e) rise. (2) Tenets of the Creativity Developmental Framework: (a) practice pathways; (b) physical literacy; (c) nonlinear pedagogy including teaching games for understanding and the constraints-led approach; (d) creative thinking. 3) Creativity training components: (a) attempts, (b) efficacy, (c) versatility, (d) originality. 4) Tactical behavior principles: (a) fundamental game principles, (b) situational game principles, (c) specific collective game principles.

Creativity development is a holistic process that underpins complex interactions among several domains (see Memmert, 2015b). In fact, the appropriate enrichment context proposed by our CDF combines a variety of training approaches embodied in creative assumptions: (a) practice pathway (from diversification to specialization); (b) physical literacy (learning of fundamental movement and game skills); (c) nonlinear pedagogy which underpins, the constraints-led approach, teaching games for understanding and differential learning; and (e) creative thinking (divergent and convergent thinking debate) (see Figure 1). Individual and integrated contribution of these approaches assures the ideal conditions that nurture and support the long-term creative development process.

## The “thinking cap” process is fertile ground for creativity

The field of creativity emerged as an outcome of the pioneering works of Joy Paul Guilford and Ellis Paul Torrance. The last one is particularly responsible for the most recognized and widely used assessments of creativity performance, such as the Torrance Tests of Creative Thinking (Cramond et al., 2005). Usually, the main topics of researchers' interest are the development of effective ways to enhance creativity through optimizing the environment (Ma, 2006; Davies et al., 2013; Onarheim and Friis-Olivarius, 2013). Despite the lack of studies, the CDF considers crucial raise a creative thinker and a technical-tactical creative player, as complementary pairs. Nurture thinking abilities allow to educate open-minded players who exploit all available creative possibilities in the field. That is, raising a thinking trait predisposition to solve game problems in an unusual way.

Over time, a number of attempts have been made to explain the main criteria of a creative product (Kaufman and Baer, 2012). Despite these efforts, the CDF embraces the most recognized description which states that creative products must be centered on two core elements: novelty and appropriateness of the action (Sternberg and Lubart, 1999). The CDF proposal underpins two wide classes of thinking processes: generate novelty (via divergent thinking abilities) and select novelty (via convergent thinking abilities) (Fürst et al., 2016). Convergent actions are considered a process which generates a possible solution to a given problem and that provide criteria of effectiveness and novelty (Colzato et al., 2013). This ability encompasses analytical processes and emphasizes the importance of critical thinking (Byernes and Dunbar, 2014). During a football game, for example, the players perceive the environmental information and select an action such as shooting the ball (convergent thinking). Their critical thinking will be reflected when analyzing their action and searching for improvements, such as the appropriateness of decision-making and the shoot execution.

**Figure 2:**
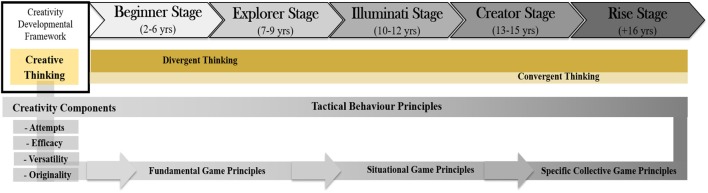
**The connection between creative thinking and the learning of tactical behavior principles (fundamental, situational and specific collective game principles) embodied in the creativity training stages**.

On the other hand, creative behavior also demands divergent abilities. Recent reports identify divergent abilities as a valid and reliable creativity predictor (Runco and Acar, 2012). Divergent thinking produces a variety of ideas and associations to a problem and forms one major cognitive process in creativity (e.g., identifying a range of possibilities to solve a problem). Precisely, creative actions emerge by the players effort to modify and produce different movement patterns generating variability (Runco and Acar, 2012; Furley and Memmert, 2015). Yet, some authors emphasize that creative abilities follow distinct patterns across development. While divergent abilities significantly decrease when not stimulated, convergent abilities remain more stable throughout the player's career (Alfonso-Benlliure et al., 2013). In fact, convergent thinking is extensively requested during the child's education. Most studies identify that children's creativity began to decline around age 6 and a creative slump further occurs at the fourth and sixth grade (Claxton et al., 2005; Kim, 2011). Nevertheless, this decline can also be explained by the increase in actions' intentionality and the decrease of imagination throughout life (Duffy, 2006; Glăveanu, 2011). Undoubtedly, the imagination serves as a precursor for creativity, as creativity uses imagination to unleash their potential. While imagination underpins things that are unreal, creativity focus on things that might be possible and never experienced before. Indeed, creativity makes the imagination feasible (Duffy, 2006). To prevent this slump, there should be a preponderance of tasks designed to develop the divergent abilities in all CDF stages as opposed to tasks that develop convergent abilities (see Figure 2). Nevertheless, this framework states for an interrelationship between both types of creative thinking since quantity does not assure quality. Indeed, problems are solved through a blend of convergent and divergent thinking.

## Components of creative thinking

To ensure the proper operationalization of creative behavior, the CDF embraces several creativity training components, which have been further adapted to the sports context (Memmert and Roth, 2007). These components allow to establish a creative game index: (a) efficacy, the ability to execute as many effective movement actions as possible; (b) versatility, identified as the ability to produce non-standard actions such as, execute different forms to pass or shoot; (c) originality, the ability to generate new and unique actions that others are not likely to produce; and finally (d) attempts, recognized as any effort to perform different actions even not-effective movements, a concept added by this work. For novice players it is extremely important left their comfort zone and demonstrate as soon as possible, the initiative to perform new forms of dribbling even if not successful (attempts). This preliminary exploratory behavior will later enhance different forms of dribbling (efficacy) and nurture the ability to adapt the dribbling in different ways (versatility), until the attainment of an unique dribbling action (originality). These components were usually used for assessing the players' creative performance through direct game observation, decreasing the weakness of creative measurements normally associated to a lack of *ecological validity (Piffer, 2012)*. In a practical view, the CDF advocates that these creativity components should be embodied in the training session's environment, mainly in the learning of tactical behavior principles (*fundamental game principles, situational game principles*, and *specific collective game principles*, see Table 1). The stretching of these components provides new insights about the key ingredients that seem to increase the probability of developing creative players and should be considered as the basis of the CDF (see Figure 2). Additionally, it is really problematic for practitioners to grasp what is really meant by creativity in sports performance (Runco and Jaeger, 2012).

**Table 1:** **Description of the tactical behavior principles used to classify the tactical game complexity in team sports (adapted from Memmert and Harvey, 2010; Mitchell et al., 2013)**.

**Principles**		**Tactical Behavior**	**Description**
Fundamental Game Principles	Attack	Dribbling attempts	Ability to express comfortably with the ball, by being able to control, and dribble the opponents in tight spaces.
		Support and orienting	Support the player in possession taking an optimal position on the playing field at right time (low risk of ball losing) and in offensive positions (movements toward opponents target direction).
		Tacking ball near goal	Transport the ball together with team mates to a finishing space.
		Attacking the goal	Moving and transport the ball with teammates into spaces between and behind the defenders in order to execute the finishing action.
		Playing together	Pass the ball to the partners quickly and in a suitable manner to the situation.
		Achieving advantage	Look for spaces in order to create numerical and positional offensive advantage.
		Using gaps	Identifying the optimal gaps to make spatial decisions to accomplish tactical tasks or match situations.
	Defense	Defending attacking players	Denying and blocking the offensive players' movements.
		Defending the goal	Collective movements of defenders to prevent/stop a shot.
		Gain the ball	Defensive actions performed by the defenders to gain the ball (e.g., interception, tackle, block, cause errors such as offside).
Situational Game Principles	Attack	Explore and improvise offensive movements	Attempts to create and use novel dribbles and moves to overcome an opponent.
		Maintaining the ball possession	Ability to maintain the ball possession, by passing the ball quickly and to a free-standing team mate allowing the ball flow without the defenders gain their possession.
		Create and use space	Moving the ball into attacking /scoring positions using pitch width /length to stay away from the defenders.
		Finalization	Team offensive actions that end with the shot to the opponent's target.
	Defense	Limiting attacking options	Preventing scoring throw the limitation of attacking options (marking key players; orienting the opposite team to the sides of the field).
		Defending space	Covering space as a defensive unit.
		Applying pressure	Defenders collective movements toward the opponent with the ball to decrease the time and space to decide.
Specific Collective Game Principles		Position specific behavior	Players' tactical mission according to each specific position (e.g., marking the forward by the central defender).
		Anticipation skills	Anticipate the opponent's actions by selecting and executing the appropriate answer.
		Tactical awareness	Players' ability to recognize opportunities, know what to do and make fast/advantageous decisions.
		Team tactical behavior	Team interpersonal coordination behavior according to the environment and opponents changes.
		Game model	Fulfill general guidelines that guide the team's players' performance.

During the framework stages arise two different types of creative expressions: P-creative (personal/psychological) and H-creative (historical) (Boden, 1996). The P-creative is internal to the player and leads to the development of individual problem-solving skills in the daily routine. Moreover, it is related with the discovery of new techniques and solutions that allows to foster the player's own personal limitations (Boden, 1996). In this way, most of the players' actions and decisions are not novel in general, just novel to them. Therefore, this personal expression is commonly performed from the *beginner* until the *rise* stages. As expected, it is important to reinforce that these initial emerging solutions are not of an expert type and they are not widely recognized in the main domain. By contrast, the H-creative is a behavior that no one ever executed before and contributes to changes in history. This type of creative expression is easier to recognize in sports and it is widely evidenced in the concepts of creativity. However, it seems that in most cases a high level of expertise it is necessary, so this outcome it will appear more consistently since the *rise* stage. Note that, these novel actions should be considered as H-creativity even if the players repeats it continually, whereas in team sports context the repeated skill never happens exactly in the same environmental conditions (Boden, 1996). The environment is unpredictable and its influence is always dependent on the player's perception and past experiences. Ordinarily, the H-creative behavior is P-creative too. In this way, the learning process should invite the players to explore instead of promotes a passive copying process. This assumption can be captured by the nonlinear pedagogy assumptions. Note that the path from P-creativity to H-creativity is not predetermined. Neither of these types of creativity occurs in timely and ordered stages (Hristovski et al., 2012), considering that the development of all players is an individualized bio-psycho-social process (see Abraham et al., 2014). In this sense, the coach needs to recognize the players' motivations in order to facilitate their development and understand that some players will face more difficulties in such creative environments. For instance, Collins et al. (2016) pointed out, how some experiences during the sport path discriminate their level of performance. As so, it is important for coaches to be aware of players' readiness to work in a creative environment.

Ordinarily, the creativity concept focus in the final outcome of a creative player (H-creativity) but before the attainment of this status occurred a developmental process (P-creativity), which is not recognized in the theoretical background. Therefore, the CDF presents a whole new concept. In the first years, *the novice creative player* must be comfortable to discover and reorganize new personal solutions, toward a continuously challenge of their self-adaptation ability. Concomitantly, should be able to move and attune outside the box under the guidance of sport environment and to solve a specific game problem in a novel, feasible, unexpected and original way by starting a single act or flowing in a collective action contributing to team success. A H-creativity is extensible described in Hopsicker (2011, p. 114), which points out: *the exceptional innovator, the highly creative players, the performer of novel moves and tactics, and the producer of imaginative strategies that tend toward competitive success by perceives and anticipates challenging situations. And also, possesses richer positional and practical knowledge of his specific sporting activity*.

Playing in a team sport does not mean that the players should give up on their individual initiative. In fact, the game quality increases as higher the player individual contribution to the team collective behavior. Likewise, the collective strategy should also have space for individual creativity. That is, each player should have both the responsibility and liberty inside the team game model that allows to develop and express his potential creative. While a rich description of creative behavior can be stated, there is still a lack of detailed information about understanding the developmental path that triggers this state.

## The maturation of world class skills

The debate about the type and the amount of practice that players should invest in across their sport careers is just beginning (Collins and MacNamara, 2011; Moesch et al., 2011; Lloyd et al., 2015; Hornig et al., 2016; Rees et al., 2016) and is really controversial. Currently, two proposed pathways in the development of sport expertise prevails: diversification and specialization. These pathways are mainly differentiated in terms of their early structured process. On the one hand, the specialization is associated to structured activities typified by an early involvement in a single sport (between 5 or 6 years of age) and coupled with high amounts of specific training—deliberate practice (Ericsson, 2014; Hambrick et al., 2014). On the other hand, diversification includes an early involvement in several sports and it is also associated to low structured activities—deliberate play (Leite et al., 2009; Côté and Vierimaa, 2014; Côté and Ericsson, 2015). Notwithstanding, the concepts of specialization and deliberate practice, as well as diversification and deliberate play should not be considered as being synonymous. This common misinterpretation leads to a narrow view, which does not allow to exploit the potential of a possible coexistence between the previous concepts (Coutinho et al., 2016). Therefore, in the *rise* stage, the players can specialize only in the main sport and being exposed to a nonlinear training method.

**Figure 3:**
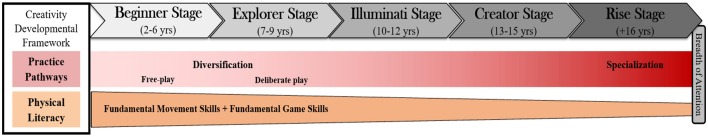
**The type of practice pathway and physical literacy structure embodied in creativity training stages**.

In this sense, the CDF recognizes that both sport pathways are not antithesis but they can coexist since they correspond to distinct moments of talent development. Moreover, the framework states that a continuum of practice activities ranging from unstructured settings, which normally guides toward divergent abilities, should precede the highly structured settings, focusing on the convergent abilities. This sequence may have an important role in the boost of creative behavior (see Figure 3).

Despite several statements referring that pursuit of a specialized pathway drains players' creativity, the expertise in a structured domain in later stages is an essential condition to be truly creative. It seems that outstanding behaviors can only be displayed if players master the field (Ericsson and Lehmann, 1999). Such preparation is an essential requirement to attain the elite status and compete at the highest level. In later stages, specialization in one sport is mandatory providing a qualitative jump to push the limits of the creative progress. However, is there an optimal stage or time to specialize in team sports? Definitely, it is hard to address this issue but several Cotê studies (Côté et al., 2009; Côté and Vierimaa, 2014) proposed the age of 16 as a suitable time (e.g., rise stage).

Additionally, expert players possess the ability to successfully combine their motor and perceptual functions during the game to outplay the opponents (Abernethy et al., 2005; Broadbent et al., 2015; MacNamara and Collins, 2015). Recently, research has established a positive relationship between creative behavior and particular aspects of these perceptual skills, such as the breadth of attention (Memmert, 2011). Usually, the breadth of attention is the term used to refer to the number and range of stimuli that a player is able to attune at any moment (Memmert, 2009). The players need a wide breadth of attention in order to generate tactical behavior and seek for different solutions (Memmert and Furley, 2007). In fact, the CDF makes it possible to associate different stimuli with each other increasing the potential to display novel game solutions (Kreitz et al., 2015; Memmert, 2015b). In a specialization phase, the lack of fundamental principles (technical and tactical) affect their ability to select the relevant environmental information decreasing the emergence of a highly adaptive behavior. Ericsson and Lehmann (1999, p. 76), summarized the connection among expertise and creativity by concluding that: “…*individuals have not been able to make generally recognized creative contributions to a domain unless they had mastered the relevant knowledge and skills in the course of a long preparatory period*.” In order to achieve that level of proficiency, players must accumulate a large amount of hours of practice. In early stages, those hours can be accumulated in non-specific activities, suggesting a functional role in the development of expertise (Abernethy et al., 2005; Leite and Sampaio, 2010; MacNamara and Collins, 2015). In addition to later specialization as a potential contributor for H-creativity development, the CDF suggests that diversification could also be a crucial element.

The concept of early diversification prior to a later restraining, typically associated to specialization, is regarded as mandatory to enhance divergent thinking (Memmert and Roth, 2007). The exposure in a variety of sport/game contexts provides invaluable experiences and stimulus for players, and enables them to adapt to unpredictable situations in a range of different environments (Memmert, 2006). Research has demonstrated the effectiveness of an early diversification approach while considering the unpredictable and demanding environment of team sports (Memmert and Harvey, 2010). Within the diversity of sport experiences, this developmental progression should also include the participation in *naive free-play* and *deliberate play* activities (Côté and Vierimaa, 2014). In fact, a growing body of research has found a correlation between play activities and creativity development (Memmert, 2011).

Nevertheless, the CDF tried to overcome a few gaps related with literature concepts. Generally, *deliberate play* is presented in literature as playing sport games informally which are extremely playfulness activities and promotes an incredibly pleasure, performed with rules adapted from adult norms that are set-up and monitored by children themselves in their free time (i.e., neighborhood pickup games and street football and basketball) (Côté et al., 2003; Hendry and Hoges, 2013). Considering the roots of the term *deliberate* it is preferred to use in this framework the term *naive free-play* for the previous concept. Nonetheless, it redirects the term *deliberate play* to an activity that is highly enjoyable designed to keep players motivated. However, it is oriented with a minor goal of improving athletes' overall sport skills and is slightly guided by adults. In terms of structure, *deliberate play* activities are usually more structured than *naive free-play* but less structured than formally organized activities such as *deliberate practice*. Still, during the *beginner* and *explorer* stage, the naive and deliberate play experiences assure the appropriate evolvement emerging the first sparks of a problem-solver player (P-creativity type) (Memmert, 2013).

Creativity is facilitated by play activities, which provide freedom to experiment with different movements and tactic variations and gives children the opportunity to discover, create and innovate their actions (Greco et al., 2010; Bowers et al., 2014; Pesce et al., 2016). This type of practice might encourage the child's ability to think divergently and convergently (Greco et al., 2010). To support these statements, research focused on the developmental path predictors of creative players showed an association between the time spent in *deliberate play* activities and the increase of creativity in team sports (Memmert et al., 2010). Also, a recent study examined if the amount of time spent in two different sport settings (structured vs. unstructured) during childhood has a later influence in general creative thinking. According to this analysis, time spent in unstructured sport activities was positively related to the achievement of creative thinking in adulthood (Bowers et al., 2014). Likewise, it is also recognized that early participation in highly enjoyable activities such as deliberate play builds a sport solid foundation with a long-term effect on young players' intrinsic motivation (Ford et al., 2009). The social learning theory supports the previous statements (Bandura, 1969), it sees individual differences in behavior as resulting from early learning experiences. According to this theory the children's first experiences in sports will later shape sporting behavior and the type of commitment. Thus, coaches should be aware that must provide a plenty of positive experiences during the early years. These challenging and pleasant environments provide the optimal conditions to nurture the children to continue their journey surrounding the fundamental abilities to improve their physical literacy. Indeed, creativity and expertise research have not been discussed in parallel. Despite the clear evidence about the benefits of the unstructured activities in creativity, the magnitude of the effect of joining them with more structured practices in later stages it is still unclear.

## Physical literacy

In the last 10 years evidence has grown regarding the importance of physical literacy (see Giblin et al., 2014; Macnamara et al., 2015). The concept describes a holistic engagement that encompasses the ability of an individual to use cognitive processes to help children move with confidence in a range of novel and challenging environments (see Longmuir et al., 2015). Mainly, at the *beginner and explorer* stages, children should be encouraged to engage with low-structure activities that promote a substantial improvement of physical literacy (Lloyd et al., 2015). In sports setting, physical literacy is often associated with fundamental movement skills, which include the mastery of locomotive (running, skipping, and hopping), manipulative or object control (catching, throwing, grasping, and striking), and stabilizing skills (balance, rotation, bracing, and twisting) (Lubans et al., 2010; Lai et al., 2014; Jaakkola et al., 2016). However, physical literacy also includes the fundamental game skills, in which children learn specific sport skills, such as how to kick the ball without an interception of the opposing team. Literate players build up easily a sustained skill background that allow them to be more creative.

Research typically emphasize the transfer hypothesis, suggesting that children should acquire fundamental movement skills before learning the related fundamental game skills (see Gallahue et al., 2011). For example, young children should be first taught throwing patterns in ways that are not sport specific before undertaking the learning of specific skills such as the basketball chess pass or handball shoulder pass. Otherwise, children may encounter more difficulties when learning complex sports skills, which may influence the pursuit of sport excellence. The breach of this principle seems to lead to a *proficiency barrier*, which could affect the progression into complex sports-specific skills being dependent on the prior foundation of fundamental movement skills (Seefeldt, 1980; Gallahue and Donnelly, 2007; Kalaja et al., 2011; Bryant et al., 2013). Moreover, the previously assumptions are slowly fading away, and the CDF suggests that this standpoint surrounding fundamental movement skills might neglect the central tenets of *physical literacy* concept (Whitehead, 2010). This framework argues that the essential embodied nature of movement learning can only be understood in relation to the environment and cannot be interpreted solely as developing fundamental movement skills, which is a very diluted and restricted statement. The postulates of the CDF emphasizes the complementarity of fundamental movement skills and fundamental game skills (Ford et al., 2011; Smith, 2014), both with equal value in all stages contributing to promote versatility and competence to foster “outside the box” thinking and acting.

Considering that physical literacy includes an ability to use cognitive processes, the technique should not be taught separately from tactics (Smith, 2014). To reach an even more effective performance, the perceptual and decision-making capacities (e.g., the perception of teammate's spatial distribution) and inter-limb coordination (e.g., shoot as we run) will be needed. In fact, passing the ball in a pre-planned way is totally different from passing the ball in response to positioning of teammates and opponents in the game. While children learning fundamental game skills, the appropriate technique will emerge as natural process of adaptive play. It can be argued that learning certain movement skills can only become meaningful when they are applied in ecological sport settings (Smith, 2014). During the *beginner* and *explorer stage*, the physical literacy training provides the proper environment for learning tactical principles, mainly the fundamental game principles (dribbling attempts, support and orienting, taking ball near the goal and attacking the goal, playing together, achieving advantage, using gaps, defending attacking players, defending the goal, and gain the ball). These principles are extensively described in the CDF (Memmert and Harvey, 2010; Mitchell et al., 2013), which nurture further excitement associated with playing team sports (see Table 1).

Indeed, the physical literacy training is considered to be the building block of sport performance and if players do not excel in the basics, they will hardly display creative behaviors (Ennis, 2015). While several theoretical developmental models suggest that the trainability of fundamental skills decreases after seven years of age, other studies extend this window at least until twelve years (*illuminati* stage) (see Ford et al., 2011). Despite the lack of studies, the framework also stresses a smaller spectrum during the *creator* and *rise* stages since physical literacy is conceptualized as a journey throughout life span (see Figure 3). Physical literacy training should be maintained in the later stages but with different extents. However, the intervention strategies in a long-term preparation still remain unanswered (Longmuir and Tremblay, 2016).

## The breakthrough of ecological constraints

Evidence from the Dynamical Systems Theory are in line with CDF assumptions. Learning theories within the dynamical systems perspective allow to respond to the challenges of acquiring functional movement behaviors in dynamic environments encountered in all sports (Edwards, 2010). Understanding how movement coordination emerges and its acquisition is a central issue. Coordination arises as many system degrees of freedom (i.e., movement possibilities) are continuously organized taking into account the interaction between learner, the task and environment (Chow et al., 2015). A three stage model was proposed to understanding how movement system degrees of freedom are continuously evolving (Bernstein, 1967; Newell, 1985). In the novice stage of learning the body is kept rigid and inflexible with restricted movements. Considering that the degrees of freedom are limited the coordination solutions are also reduced. Over the course of learning, the advanced stage of learning is related with a releasing of the degrees of freedom toward more functional, fluid, and smoothly coordinated movements. This stage is characterized by the freeing of movement possibilities called synergies as the individual learn how to control and coordinate complex movements. Gradually, through this exploratory process, the learners' moves into the expert stage of learning marked by the perception of the environment interaction. The learner is able to reorganize the degrees of freedom in order to reach the most efficient and flexible movement patterns. The information available in the performance environment is crucial in shaping how learners reorganize their individual motor system degrees of freedom (see Chow et al., 2015). The manipulation of tasks constraints can shape the learner's degrees of freedom, since the changes in movement patterns leads to a transition from one stable state of motor organization toward another (Edwards, 2010). In this sense, acquisition of coordination is an ongoing process. Still, this approach may have a distinct impact on different learners, as the acquisition of movement coordination is not a linear process.

**Figure 4:**
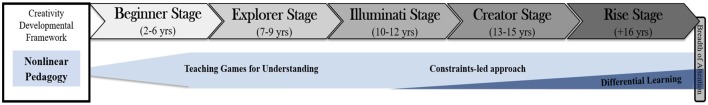
**The nonlinear pedagogy assumptions embodied in the creativity training stages**.

Due to the dynamism and nature of complexity of team sports, the CDF structure was grounded in nonlinearity (Davids et al., 2003; Harbourne and Stergiou, 2009; Chow et al., 2011; Vilar et al., 2012; Lebed and Bar-Eli, 2013; Gordon, 2014) from the *beginner* until the *rise* stage (see Figure 4). Chow and colleagues proposed the term of Nonlinear Pedagogy, a constraints-led approach (Chow et al., 2007). Nonlinearity is the basis of multistability, which is defined as the existence of more than one solution to a goal and is regarded as a crucial element for the divergent training upgrading (Hristovski et al., 2011; Memmert, 2015b). Usually, the Nonlinear Pedagogy is putted in practice through the constraints-led approach which underpins the designed principles emanating from an ecological dynamics standpoint and provides a fundamental theoretical impetus for practitioners to thrive the creative tactical behavior: (a) manipulation of constraints; (b) functional variability; (c) attentional focus (d) representativeness, and (e) the maintenance of pertinent information-movement couplings (Chow, 2013; Chow et al., 2015). Firstly, the role of constraints has been put forth as a central tenet encouraging the emergence of functional movement solutions within game situations (Lee et al., 2014). Typically, task constraints can be readily manipulated to boost the discovery and exploration movement solutions (Hristovski et al., 2011). The associated variability in task performance inspires players to adapt and unlock their creative potential. Indeed, the *noise* provided by these challenging environments promote the exploratory behavior (Schollhörn et al., 2009) and players will be channeled to functional and versatile connections that may lead them to creative behaviors. The CDF reasons that the exploratory behavior is a central tenet and a precondition of player's creative development that should persist until the *rise stage* with different magnitudes. Moreover, giving opportunities to young players to explore their boundaries is fundamental, mainly in *explorer* and *illuminati stages*, making them more adaptive without being afraid to attempt and risk-taking in competitive situations. Players need the opportunity to search for their own task solutions instead of applying standardized responses. Therefore, the use of representative environment training situations seems to be the way in which players become more proficient at perceiving environment cues and constant changes in game situations. In this sense, the use of more ecological training situations allow players to attune relevant sources of information based on information-movement coupling. Passing in football, for example, should be developed with an opponent attempting to intercept the ball (perform under defensive pressure instead of passive or without defenders) and under variable task constraints (game rules, space and equipment change). Moreover, a constraints-led perspective can be used to sustain the design of training tasks across the learning styles. Concepts derived from the Mosston's Spectrum of Teaching Styles (Moston and Ashworth, 1990) reinforce the physical literacy and nonlinear previous statements. This theory comprises a spectrum moving from teacher-centered to student-centered styles. The further players-centered styles of the spectrum, are the most suitable for developing creative players (problem solving and creativity styles) (Sicilia-Camacho and Brown, 2008). In the field of sports, the Teaching Games for Understanding (TGfU), as a game-centered pedagogy fits perfectly with these problem solver styles.

Over the last twenty-years, research on popular pedagogical approaches such as the *TGfU* has been increasing in scientific literature (Butler, 2014). The focus of *TGfU*, which incorporates key aspects of Nonlinear Pedagogy principles, is to provide experiences centered in technical and tactical skills through modified game contexts (Tan et al., 2012; Butler, 2014; Harvey and Jarrett, 2014). Additionally, it favors the game understanding (e.g., critical-thinking and problem solving skills), since technical and tactical skills are developed simultaneously, clearly linked with the physical literacy's previous standpoints. Farther, the TGfU approach seems to be a suitable way to enhance physical literacy (Mandigo and Corlett, 2010). The framework considers these modified games applied in ecological situations, the fingerprint of the *TGfU* approach and the main driver for the tactical creative behavior (Memmert and Harvey, 2010). Nevertheless, the main critic pointed out to the TGfU approach is related with the progressive loss of the coaches' responsibility by simply allowing players to play games without any criteria. Indeed, designing representative practice tasks can be more time consuming and demanding for coaches (Memmert et al., 2015; Renshaw et al., 2015).

In order to promote tactical creativity and to better understand how to manipulate the task constraints, the *TGfU* model embraces four key pedagogical principles strongly supported by core principles of nonlinearity: sampling, modification-exaggeration, modification-representation, and tactical complexity level (see Tan et al., 2012; Chow et al., 2015), these are usually implemented in Small-Sided Games (Hill-Haas et al., 2011; Travassos et al., 2012; Halouani et al., 2014). Games with less tactical complexity should be taught first, during the *explorer* and *illuminati* stages, to ensure that novice players understand the game they play. It is important to consider that in early stages the constraints should be relaxed to enhance the exploratory behavior that consequently leads to a better efficacy and versatility of players actions (Hristovski et al., 2011). For these reasons, the CDF sustains that it is reasonable to start with simple game formats before raising tactical complexity, in *creator* and *rise* stages, by introducing Small-Sided Games with higher collective demands (Tan et al., 2012). In turn, as the game skills are mastered, the tactical complexity should be increased toward a proper adaptive response (Davids et al., 2013). The importance of high task complexity has been widely explored in the organizational field and it seems that creative people, with a certain level of expertise, tend to be attracted to complexity (Chae et al., 2015). To attend this issue, the CDF will use different levels of game complexity which gradually increase the tactical creative behavior adapted from previous works of Memmert and Harvey (2010), Mitchell et al. (2013) (see Table 1). Considering the decision-making models, in the nonlinear pedagogy approach predominates a naturalistic decision making, which result in a deeper understanding of the team member's contributions and the role of the environment in specific situations. This will enhance the collective synchrony toward a more effective play, mainly during complex situations (Macquet, 2009; Richards et al., 2012).

The CDF links the constraints-led (learner-environment) and TGfU (learner-centered) approaches in order to sustain the creativity development in team sports. Despite both using the core principles of nonlinear pedagogy as a theoretical background, it is important to make clear that they are slightly different (see Renshaw et al., 2015). These differences are harmoniously aligned allowing to stablish new conceptual insights. The key similarities include: (a) their holistic perspective of the learner; (b) consider the individual differences of the learner; (c) coach act as a facilitator to guide players' discovery; and (d) learning tasks design are sustained on the common ideas of representativeness (Renshaw et al., 2015). This framework also intends to provide clarification for sport stakeholders on the nature of the synergies that emerge within the dissimilarities between the two approaches. According to the framework the *TGfU* vision and proposals are of particular value until the *illuminate* stage. Significant increase of tactical creativity were confirmed in children among 7–10 years of age (Memmert, 2011), possibly indicating the optimal window of trainability. In fact, the TGfU is a more educational related approach designed for children to understand the generic tactical concepts of team sports and to capture a passion for playing games, clearly linked with previous standpoints of diversification and physical literacy. Likewise, the use of questioning as a main pedagogical tool toward a more critical thinking player. It seems that this approach captures these features better than the constraints-led approach, which consist in an advantage in supporting a wide-range creativity environment in early stages. Instead, the aim of the constraints-led approach is to reach the task goal taking into consideration the several possible ways of attaining the final outcome. This approach is more geared to performance and supports players to become autonomous of coaches' feedback and instructions, thus more indicated from the middle stages of the framework (from *illuminati* stage).

Further, as previously referred the TGfU sustains a simple to complex progression and during the early stages could simplify and boost the players learning. At this point, both approaches are distinct since constraints-led stages are not sequential (Chow et al., 2007). However, in later stages, given the players already have a sustained sport background this nonlinear process is crucial in the exploratory behavior. On the other hand, the TGfU approach also advocates taking students out of games to develop skills (Butler, 2014). In this point, nonlinear minor adjustments are necessary. Thus, to address this issue, the framework reinforces that a few TGfU designs based on the principles of nonlinearity would possibly lead to the creation of more effective games. For example, creating 1vs1 or 2vs1 situations instead of removing the player from the game environment. In middle and later stages (*creator* and *rise* stages), the constraints-led approach should prevail in its fullness. Note that there are minor differences among the two approaches that have not been addressed, since they have a trivial contribution to the framework.

## Differential learning

Building on current nonlinear pedagogies, differential learning explores the fluctuations, and adaptive mechanisms in perception-action coupling through performing complex movements without repetitions and adding permanent stochastic perturbations in tasks (Schollhörn et al., 2009; Schöllhorn et al., 2012). Specifically, this approach proposes infinite variations in technique movement to make the player ready to deal with disturbances in competitive environments (Frank et al., 2008; Schöllhorn et al., 2012). According to the current standpoint the variation and adaptability provided by the combination of different motor patterns allow players to better reorganize the knowledgeable skills and learn new patterns to produce a variety of novel motion configurations. In this sense, the players should be challenged in unfamiliar environments with maximal pattern variations of the initial situation, for example, the players are instructed to perform movement errors instead of avoiding them (e.g. kick a ball with arms raised) (Schöllhorn et al., 2006). In fact, differential learning provides a highly improvisation demand, one of the most complex forms of creative behavior. Research refers that improvisation is a learnable skill that requires a considerable amount of training (Beaty, 2015). Besides, the framework argues that training for improvisation is associated with a releasing effect, appreciated in creative behavior (Sawyer, 2000; Fink and Woschnjak, 2011; Kleinmintz et al., 2014). Thereby, the CDF suggests that the differential learning volume should have a significant increase starting at the *illuminati* stage and should attain their peak of prominence in the *rise* stage (see Figure 4). Differential learning should be considered with caution when it is used with young players, it seems that too much noise can slow the learning process. Nevertheless, the sport stakeholders should be aware of the differential learning applicability. Thus, this approach should be progressively introduced as the players develop their sporting fundamentals. In the *illuminati* and *rise* stages the players have already gathered an optimized technical-tactical and perceptual background as well as a greater game purposiveness.

## Summary and implications

To ensure a proper creative developmental process, the first developmental stages should be grounded on intrinsically motivating and low structured experiences, such as the involvement in deliberate play activities instead of high controlled activities. However, the transitioning from *diversification* (where prevails P-creativity type) to *specialization* (possibly an H-creativity type) should gradually occur at appropriate developmental times and players need to be correctly guided during this continuous change of sport demands. The central tenets of diversified practice leads to an enrichment of the physical literacy. The mastery of several motor and game skills during the *beginner* and *explorer* stages, guides players to move outside the box, to think critically and improve their breadth of attention to conveying knowledge to make creative connections among several contexts. Furthermore, the Framework sustains that creativity can only be requested if the environment request creativity. For this reason, players should be prepared for the next unpredictable action, which will imply the permanent challenge of adaption mechanisms. The ultimate achievement of tactical creative behavior is grounded in the nonlinear pedagogy, which is an important way to facilitate the emergence of novel and functional solutions through adaptive movement patterns. The manipulation of tasks constraints it is extremely important to promote randomness in player's actions. Moreover, this dynamical changes develops exploratory behavior that encourage players to discover new action possibilities (improving divergent thinking). In fact, the environmental information in team sports changes instantaneously, therefore it is extremely important for the players to develop their ability to attune the optimal affordances under representative learning environments.

Additionally, the constraints-led approach and TGfU is a meaningful tool for players to perceive the *affordances* during the game performance. Not devaluating the role of cognitive explanations, the CDF stresses the relationship between perception and action in the movement systems, which clarify how the learning occurs. Coordination emerges as learners adapt their movement behaviors to the constraints. In this sense, the creativity training process should focus on simulated versions of major games using small-sided games sustained in several types of constraints. This progression improves the tactical behavior learning, which must be grounded in creativity training components (attempts, efficacy, versatility, and originality). In later stages, the tactical creative behavior also needs to be supported by complex patterns skills acquisition through the differential learning approach and with a specialized commitment. These approaches makes the players more able to adapt against environment disturbances and preparing them to perform novel configurations during the game. Thus, the CDF stresses that players should be free to explore the possibilities unhindered and create without limits throughout all the developmental stages.

Overall, the CDF scrutinizes the key directions that lead to a sustainable development of the creative behavior according to age and expertise in team sports (see Table 2). In addition, this article might act as a starting point to understand creativity training, which should be gradually implemented in sports structures and physical education lessons. It was also highlighted the unpredictable environment in which team sports occur and the implications for the design of creativity long-term programs. During the developmental stages, the players will express different types of creativity (from P-creativity to H-creativity) or they even may never display the H-creativity, but certainly in the end they will be more adaptive and functional. As previously mentioned, the development of all players is an individualized bio-psycho-social process. Thus, coaches should not to wait until the complete skill development, otherwise they need to recognize and accept the limits in cognitive and motor maturity that may restrain the creative performance. Coaches should inspire players through shaping their environment. Nevertheless, we can be sure that we are on track if players feel comfortable to think differently without fear of failure, or of take risks, explore new behaviors and be innovative during the game. In this regard, the main concern of the CDF is how it will be understood and applied by coaches, teachers and practitioners. Considering that there is a substantial individual variation in creative development the presented stages are general guidelines, so they may not be sequential. During the course of all stages, many external factors, which are not mentioned interact and can interfere with the process. Still, the framework assumes that all players eventually will follow the same sequential pathway from *beginner* to *rise* stages and this assumption is not always fulfilled. Moreover, the framework only provide the overall early guidelines that should be followed in the clubs or schools, but the creative process continues beyond the *rise* stage. While the players' expertise increase, the likelihood of H-creativity appearance is higher and often occurs after the *rise* stage. In this sense, it would be necessary to incorporate later stages. However, considering the different purposes and structures of professional sports, could be extremely difficult to uniform the guidelines to be followed. Thus, the current theoretical framework should be considered as a preliminary guideline to support the coaches and teachers' understanding of creativity development issues in early stages, which is urgently required. Having this assumption in mind, it was suggested that sports stakeholders should support and adapt their training programs to embrace the players' creativity. Finally, this theoretical framework should be considered as a work in progress that offers a substantial potential for enhancing creative behavior.

**Table 2:** **Creativity stages of the Creativity Developmental Framework in team sports**.

**Creative Stages of the Creativity Developmental Framework in Team Sports**				
***Beginner*** **(2–6 yrs)**	***Exploratory*** **(7–9 yrs)**	***Illuminati*** **(10–12 yrs)**	***Creator*** **(13–15 yrs)**	***Rise (*****+16 yrs)**
*The sparks of confidence and competence to move…*	*Time to explore and pursue new solutions!*	*Moving and attuning outside the box…*	*The sports environment guides the problem solver actions…*	*All prepared, to the collective behavior spawns!*
**PRACTICE PATHWAY**				
- Naïve-Free play in a wide range of diversified contexts.	- Practice several sports embodied in *deliberate play* activities.	- Keep practice and explore several sports skills.	- Narrow gradually the diversified structured practice exposure.	- Specialization in one or two sports with a nonlinear commitment.
**PHYSICAL LITERACY**				
- Acquiring confidence and competence to move through learning fundamental movements and game skills.	- Continuous improvement of fundamental movements and game skills.	- Mastery of the fundamental movements and game skills.	- Performing complex movement skills and perceived the game *affordances*.	- Development of the creative tactical behavior.
**NONLINEAR PEDAGOGY**				
- The first experiences in invasion play-type activities. This stage is characterized by the restricted degrees of freedom (novice stage of learning).	- Embodied in representative learning contexts and in simpler modified-games forms which increase the technical background. Player moves from a novice to advanced stage of learning with a progressive releasing of the degrees of freedom.	- Embodied in representative learning contexts with moderate modified-games forms, that increase the technical and tactical repertoire. More functional, fluid and smoothly coordinated movements.	- Embodied in representative and differential learning contexts with complex modified-game forms, toward the increase of technical and tactical performance. Player learn how to control and coordinate complex movements.	- Embodied in representative learning with a special focus on differential contexts and highly complex modified-game forms that mostly increase the tactical awareness and technical configurations. Nurturing the environment interactions (expert stage of learning).
**-TACTICAL BEHAVIOR PRINCIPLES**				
- Fundamental game principles learning.	- Improvement of the fundamental game principles.	- Introduce situational game principles training.	- Situational game principles mastery.	- Moving to the specific collective game principles knowledge.
**CREATIVE THINKING**				
- Divergent thinking development through enhancing the player's curiosity traits.	- Divergent thinking development through fostering the player's exploratory technical-tactical behaviors. The P-creativity prevails.	- Divergent thinking improvement through stretching the player's exploratory technical-tactical behaviors. The P-creativity prevails however, it is possible to occur the H-creativity type.		- Divergent thinking mastery through high player's improvisation behavior. The possibility of occurring H-creativity behaviors gradually increases.
**- CREATIVITY COMPONENTS**				
- Encourages the player's attempts.	- Focus on the player's actions efficacy, without discouraging their different attempts in all stages.	- Focus mainly on the efficacy and versatility of players actions without discouraging their different attempts.	- Focus on efficacy, versatility and originality of player's actions.	- Focus mainly in efficacy, versatility and originality of players' performance.

## Author contributions

SD has been involved in drafting, conception, and design of the manuscript, developed the draft of the creativity developmental framework, combined the training approaches with creative assumptions, wrote the general topics of the article and agreed with all aspects of the work. DM developed a red line for the developmental framework with the important aspect age, ensuring that the ideas presented in manuscript were appropriately investigated and articulated with creativity topic, upgraded the creativity developmental framework, revised it critically for important intellectual content, gave final approval of the version to be published and agreed with all aspects of the work. JS carried out the drafting, conception and design of the manuscript, analysis and interpretation of the approaches, upgraded the creativity developmental framework, wrote the introduction and revised it critically for important intellectual content and agreed with all aspects of the work. NL has been involved in drafting, conception, and design of the manuscript, wrote the implications, revised it critically, also gave final approval of the version to be published and agreed with all aspects of the work.

### Conflict of interest statement

The authors declare that the research was conducted in the absence of any commercial or financial relationships that could be construed as a potential conflict of interest.
